# Making wildlife welcome in urban areas

**DOI:** 10.7554/eLife.41348

**Published:** 2018-10-02

**Authors:** Travis Gallo, Mason Fidino

**Affiliations:** 1 Urban Wildlife InstituteLincoln Park ZooChicagoUnited States

**Keywords:** mammals, urban wildlife, biodiversity, urbanization, Other

## Abstract

Careful design of the green spaces in cities will benefit both wild animals and humans.

**Related research article** Parsons AW, Forrester T, Baker-Whatton MC, McShea WJ, Rota RT, Schuttler SG, Millspaugh JJ, Kays R. 2018. Mammal communities are larger and more diverse in moderately developed areas. *eLife* **7**:e38012. doi: 10.7554/eLife.38012

Can we think of a world in which wild animals wander down our streets? It may be difficult to picture such a scene because cities have historically been designed and built with people, rather than biodiversity or wildlife, in mind. And as urban areas grow – it is expected that 95% of population increase over the next decade will be in cities (Acuto et al., 2018) – it becomes even harder to conceive that humans and wildlife could co-exist in urban landscapes. However, there are some grounds for optimism and now, in eLife, Arielle Parsons of the North Carolina Museum of Natural Sciences and North Carolina State University, and colleagues report new insights about the wildlife that live in cities (Parsons et al., 2018).

The team used remotely triggered wildlife cameras in two cities – Washington, DC and Raleigh, North Carolina – to compare mammal diversity and habitat use across a range of environments: urban, suburban, exurban (i.e., commuter towns beyond the suburbs) and wild spaces. While the diversity was low in the most urban areas, there was little difference in the number of species and the habitat use of wild mammals between suburban areas and more natural settings. Most of the animals that lived in the wider region were also found in suburban areas, thus offering new insights in the ways that wildlife can co-exist with humans in an urbanizing world.

However, the suburbs of today are likely to be the urban areas of tomorrow. In Chicago for instance, the human population is expected to grow by another 2.4 million people by 2040, with suburban areas absorbing 64% of that increase. As suburbia becomes densely populated, what can be done to protect biological diversity? Parsons et al. point to the answer: in their study, mammals were more likely to occupy areas with higher levels of urban green space. This result adds to a growing body of research highlighting that these green areas are important habitats for biodiversity (Figure 1; Aronson et al., 2014; Aronson et al., 2017; Ives et al., 2016; Hall et al., 2017).

**Figure 1.**
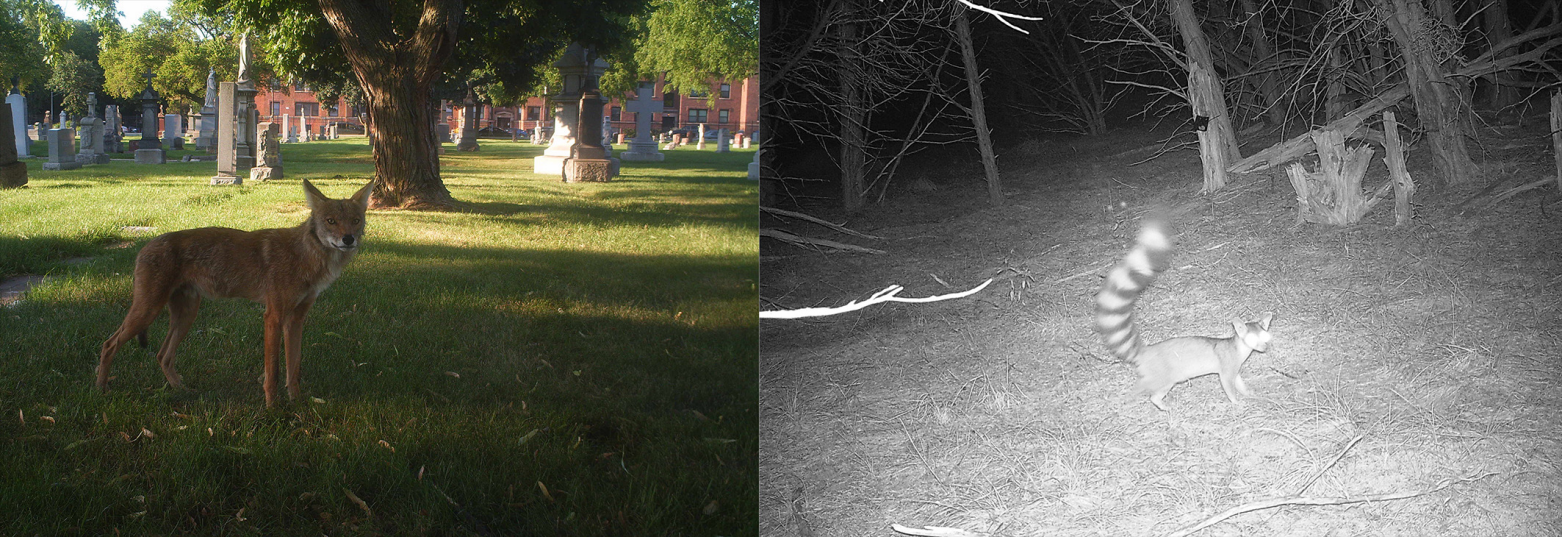
Wildlife can persist in urban areas when habitats are avaliable. A coyote (*Canis latrans; *left) captured on a trail camera in a cemetery in the Uptown neighborhood of Chicago, Illinois. A ringtail (*Bassariscus astutus*; right) captured on a trail camera near downtown Austin, Texas. Parsons et al. studied wildlife in two cites – Washington, DC and Raleigh, North Carolina – and found that, as the human population density increased, the amount of green spaces available became more important for urban wildlife. Photographs: Urban Wildlife Institute, Lincoln Park Zoo; Amy Belaire.

Yet, not all the green spaces in cities are created equal for wildlife. For example, with colleagues we have shown that urban parks in Chicago, with their mature trees embedded in a sea of turf grass, are relatively poor habitats for mammals. In comparison, cemeteries or golf courses, which often are surrounded by additional vegetation and have open water areas, hosted more species (Gallo et al., 2017). Understanding what makes certain urban green spaces friendly to wildlife can help us to design parks that attract a wider range of animals. Ultimately, people also benefit from city parks, which purify the air, cool the temperature, and provide a space to engage in physical activity that can increase well-being and reduce chronic diseases like childhood obesity (Wolch et al., 2014).

Another compelling aspect of the study by Parsons and colleagues – who are based at universities and institutes across the United States – is that the results came from a large-scale citizen science project, where 557 volunteers operated 1427 cameras in their own backyards or in green spaces near their homes. While urban ecologists and conservation scientists primarily study the ecological form and function of cities, many practitioners also hope to connect urban dwellers with their natural surroundings. Experiences explicitly focused on biodiversity – such as citizen science projects – help people develop a greater global conservation ethic compared to schemes like community gardens, where biodiversity is raised more implicitly (Prévot et al., 2018). Although Parsons et al. did not measure the outreach and stewardship potential of their work, it is possible that the study increased the ecological knowledge of urban and suburban residents, deepening their understanding of global conservation issues and having a broader impact on urban conservation than expected.

Cities create near permanent changes to the landscape and they can seriously damage global biodiversity. However, as scientists like Parsons and colleagues show, nature can – and does – find a way to adapt to urban spaces. Therefore, by taking a social and ecological approach to urban planning, we can create environments that see people thrive, and wild animals roam.
